# The Role of FGF1 in Chronic Liver Diseases

**DOI:** 10.3390/biomedicines14071436

**Published:** 2026-06-24

**Authors:** Tao Liu, Meihong Yu, Liu Han, Jing Wu, Deliang Liu, Yuyong Tan

**Affiliations:** 1 Department of Gastroenterology, Second Xiangya Hospital, Central South University, Changsha 410011, China; taoliucsu@163.com (T.L.); 2204130510@csu.edu.cn (M.Y.); hanliu8371@163.com (L.H.); 18821928047@163.com (J.W.); 2 Research Center of Digestive Disease, Central South University, Changsha 410011, China; 3 Clinical Research Center for Digestive Diseases in Hunan Province, Changsha 410011, China; 4 Guilin Hospital of the Second Xiangya Hospital, Central South University, Guilin 541002, China

**Keywords:** fibroblast growth factor 1, chronic liver disease, drug-induced liver injury, metabolic dysfunction-associated steatotic liver disease, alcohol-related liver disease

## Abstract

Chronic liver disease (CLD) constitutes a major global health burden, with high morbidity and mortality, limited treatment options for several etiologies, and an urgent need for novel therapeutic targets. Fibroblast growth factor 1 (FGF1) is a unique member of the FGF family capable of binding all four FGFR subtypes, thereby regulating multiple signaling pathways including PI3K/AKT, Ras/MAPK, and PLCγ, which are involved in metabolism, cell survival, proliferation, and tissue repair. Emerging evidence highlights the multifaceted and context-dependent roles of FGF1 in CLD. In drug-induced liver injury (DILI) caused by anti-tuberculosis drugs, acetaminophen, or doxorubicin, FGF1 confers protection by restoring bile acid homeostasis, reducing oxidative stress, inflammation, and apoptosis. In Metabolic dysfunction-associated steatotic liver disease (MASLD), FGF1 ameliorates hepatic steatosis, oxidative injury, and insulin resistance through downregulation of SREBP1, upregulation of PPARα, and activation of Nrf2-mediated antioxidant responses. Conversely, in primary sclerosing cholangitis (PSC), FGF1 aggravates ductular reaction, biliary senescence, and liver fibrosis via upregulation of SASP and TGF-β1, suggesting that inhibition of the FGF1/FGFR axis may be therapeutic. For alcohol-related liver disease (ALD), although direct experimental evidence is lacking, FGF1 is hypothesized to confer protection given its known activities against oxidative stress, lipid dysregulation, and cell death. Despite its promise, the mitogenic potential of FGF1 raises safety concerns; however, N-terminally modified FGF1 analogs (e.g., FGF1Δ) retain metabolic benefits with reduced proliferative activity. Collectively, FGF1 represents a versatile and disease-dependent regulator in CLD, warranting further mechanistic studies, safety evaluations, and development of targeted analogs as a novel therapeutic strategy for difficult-to-treat liver diseases.

## 1. Introduction

Chronic liver disease (CLD) represents a major global public health challenge, imposing an enormous social and economic burden with high morbidity and mortality worldwide. Approximately 2 million people die from liver diseases each year [1], and the quality of life of affected patients is severely impaired, accompanied by increased risks of various complications, heavy medical expenses, and significant loss of disability-adjusted life years [2]. Among those aged 25 to 49, liver diseases are the 12th leading cause of disability-adjusted life years, and this potential loss of life is even higher in developed regions like Europe [3,4]. Historically, hepatitis virus infection has been the primary cause of CLD, but with the popularization of vaccination and the application of targeted therapeutic drugs, its proportion is gradually declining. Instead, drug-induced liver injury (DILI), alcohol-related liver disease (ALD), Metabolic dysfunction-associated steatotic liver disease (MASLD), and primary sclerosing cholangitis (PSC) have emerged as the predominant etiologies of CLD in many developed areas, and some of these diseases still lack specific and effective targeted therapeutic drugs [1,5,6,7]. The complex pathogenesis of CLD involves multiple pathways including oxidative stress, lipid metabolism disorder, mitochondrial dysfunction, immune response abnormality, and bile acid homeostasis imbalance, highlighting the urgent need to explore novel regulatory molecules and therapeutic targets for the development of effective intervention strategies.

**Figure 1:**
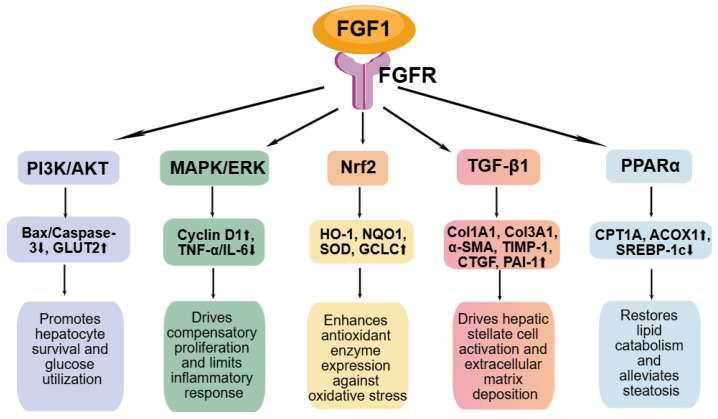
Schematic illustration of FGF1-mediated multi-pathway regulation in chronic liver diseases.

Fibroblast Growth Factor 1 (FGF1), also known as acidic FGF (aFGF), is a small polypeptide with a molecular weight of approximately 17–18 kDa. Distinguished from classic secretory proteins, FGF1 lacks a typical secretory signal peptide and is secreted by cells into the extracellular space via a paracrine pathway, exerting biological functions by binding to fibroblast growth factor receptors (FGFR) and heparan sulfate proteoglycan as an auxiliary factor [8,9]. Uniquely, FGF1 is the only protein in the FGF family that can bind to all four FGFR subtypes (FGFR1 to FGFR4), enabling it to activate multiple downstream signaling pathways and regulate diverse physiological and pathological processes (Figure 1) [10,11]. Through activating the phosphatidylinositol 3-kinase (PI3K)/AKT pathway, FGF1 modulates metabolic regulation, glucose uptake, and cell survival [12]. By activating the Ras/mitogen-activated protein kinase (MAPK) pathway, FGF1 promotes cell proliferation and differentiation, and supports tissue repair and metabolic regulation [13,14]. Via the phospholipase C γ (PLCγ) pathway, FGF1 promotes intracellular calcium signal transduction and activation of protein kinase C (PKC), thereby regulating gene expression and cellular metabolism [15]. Additionally, FGF1 regulates cell migration, differentiation, proliferation, apoptosis and wound healing by increasing the phosphorylation level of STAT3 [16]. As a crucial metabolic regulatory molecule, FGF1 is widely expressed in various tissues of the body, and accumulating studies have demonstrated its important regulatory roles in the occurrence and development of multiple CLDs, either exerting protective effects by alleviating liver injury or participating in disease progression by regulating biliary response and fibrosis, making it a potential therapeutic target for CLD.

## 2. The Role of FGF1 in Drug-Induced Liver Injury

Drug-induced liver injury (DILI) refers to liver damage caused by drug exposure. Its clinical manifestations and pathological mechanisms vary with drug characteristics, dosage, exposure duration, and host-specific factors [17]. The identified mechanisms include mitochondrial dysfunction, excessive reactive oxygen species (ROS) production, hepatocyte apoptosis/necrosis, and immune-mediated bile duct damage [18]. The etiologies of DILI exhibit regional differences: antiretroviral drugs, anti-tuberculosis drugs and herbal medicines are the main causes of DILI in Asia and Africa, while antibiotics, non-steroidal anti-inflammatory drugs and psychotropic drugs predominate in Europe and the United States [19].

The long course of tuberculosis and the hepatotoxicity of anti-tuberculosis drugs have made anti-tuberculosis DILI one of the most common types of DILI [20]. The risk of liver toxicity for first-line anti-tuberculosis drugs, such as isoniazid and rifampicin, ranges from 2% to 28% [21], which may lead to adjustments or interruptions in the anti-tuberculosis treatment and can even cause acute liver failure and even death in severe cases [22]. The mechanisms of hepatotoxicity associated with isoniazid and rifampicin include reduced bile acid transporters, leading to bile acid accumulation and disruption of the Bcl-2/Bax balance in hepatocytes, release of cytochrome c, and triggering of cell apoptosis [23]. Lin et al. found that isoniazid and rifampicin significantly increase serum total bile acid levels and decrease the liver FGF1 expression levels in mice, and exogenous FGF1 administration can restore the bile acid homeostasis and alleviate the liver damage [20]. Further research has revealed the FGF1 can act as a negative regulator of bile acid synthase, inhibiting its expression through the FGFR4-ERK1/2-HNF4 signaling pathway [20].

Acetaminophen, a widely used analgesic and antipyretic, can cause severe liver damage or even death [24,25]. Excessive acetaminophen leads to the formation of excessive NAPQI in the liver, which depletes large amounts of glutathione and forms toxic protein adducts, further inducing mitochondrial dysfunction, oxidative stress, inflammation, and DNA damage [26,27,28,29]. Under conditions of acetaminophen overdose, mouse serum ALT and AST levels significantly increased, with severe inflammation, apoptosis, oxidative stress, and endoplasmic reticulum stress observed in hepatocytes, including elevated inflammatory factors IL-6 and TNF-α, upregulation of pro-apoptotic protein Bax, and downregulation of anti-apoptotic protein Bcl-2. Injection of FGF1 could reverse these pathological changes [30]. Currently, N-acetylcysteine is the only approved antidote for acetaminophen overdose, but its treatment window is relatively short, making FGF1 a promising alternative intervention strategy [31].

Doxorubicin (DOX), a broad-spectrum anti-tumor drug, directly damages the liver (the main organ involved in the metabolism and detoxification) through ROS-induced oxidative stress [32,33]. The use of DOX leads to decreased body weight and liver weight, increased ALT and AST in mice, which is more severe in FGF1 knockout mic [34]. FGF1 deficiency reduces Nrf2-mediated antioxidant gene expression, increases 3-nitroso modification of various proteins and liver malondialdehyde content, further exacerbating oxidative stress and liver damage [34]. Exogenous FGF1 administration can alleviate DOX-induced liver injury, inflammation, and hepatocyte apoptosis, and can synergize with resveratrol to enhance its protective effect against liver injury [35].

Given the limited clinical treatment options for DILI (mostly only drug withdrawal) [17], FGF1 is expected to become a precise intervention target for DILI therapeutic drug development.

## 3. The Role of FGF1 in Metabolic Liver Diseases

Metabolic dysfunction-associated steatotic liver disease (MASLD), formerly known as non-alcoholic fatty liver disease (NAFLD), has undergone a name change that highlights the bidirectional interaction between fatty liver and metabolic changes, and emphasizes that its onset excludes alcohol consumption and other independent liver disease factors [36]. MASLD is the most prevalent metabolic CLD, affecting approximately 25% of the global population, with a projected increase to 33.5% of adults by 2030 [37,38,39]. It is characterized by hepatic steatosis accompanied by at least one cardiometabolic risk factor, such as obesity, type 2 diabetes, hypertension or dyslipidemia. Its core pathogenesis involves lipid metabolism disorder, mitochondrial dysfunction, oxidative stress, insulin resistance, and hyperinsulinemia [40]. FGF1 exerts a multifaceted protective effect on MASLD through regulating these key pathological links.

Dysregulation of liver lipid metabolism can lead to hepatic steatosis resulting from disrupted balance between fatty acid uptake, fat synthesis, oxidation, and output which results from hepatic steatosis. Fat synthesis is mainly regulated by the transcription factor SREBP1, which promotes lipid accumulation by regulating the expression of downstream fatty acid synthase (FAS), stearoyl CoA Desaturase-1 (SCD-1) and other fat synthesis-related enzymes [41,42]. Fat oxidation is regulated by peroxisome proliferator activated receptor alpha (PPARα), which enhances fat oxidation by activating genes involved in mitochondrial, peroxisome, and endoplasmic reticulum lipid oxidation, and thereby avoiding excessive liver fat accumulation [43]. FGF1 treatment of MASLD mice can effectively reduce hepatic steatosis, and serum AST and ALT levels by downregulating SREBP1 expression and upregulating PPARα expression, thereby weakening fat synthesis and enhancing fat oxidation [44,45].

Mitochondrial dysfunction plays an important role in MASLD progression. Excessive lipid accumulation induces mitochondrial dysfunction, impairing energy production, reducing oxidative capacity, and increasing ROS generation [46]. These ROS can further damage mitochondrial β-oxidation function and consume mitochondrial antioxidants such as glutathione, thereby exacerbating mitochondrial damage and forming a vicious cycle [47,48]. In MASLD mice, FGF1 treatment significantly promotes Nrf2 nuclear translocation, and increases the expression of downstream antioxidant enzymes such as NAD(P)H dehydrogenase, counteracting oxidative damage caused by lipid overload [45].

There is a bidirectional relationship between MASLD and metabolic diseases: obesity and diabetes patients have a higher prevalence of MASLD, and vice versa [49]. Improving metabolic diseases plays an important role in the treatment and prognosis of MASLD [36]. FGF1 has been reported to improve lipid metabolism and maintain glucose homeostasis in diabetes and obesity in recent years [50,51]. FGF1 improves insulin resistance by increasing insulin receptor substrate 1 (IRS1) phosphorylation, phosphatidylinositol 3-kinase (PI3K) pathway, and promoting translocation of type 4 glucose transporter (GLUT4) to the cell membrane, thereby enhancing cellular glucose uptake, and maintaining glucose homeostasis [52,53]. Collectively, the multifaceted protective effect of FGF1 makes a potential target for MASLD treatment.

## 4. The Role of FGF1 in Primary Sclerosing Cholangitis

Primary sclerosing cholangitis (PSC) is a progressive chronic cholestatic CLD characterized by intrahepatic and/or extrahepatic bile duct injury, manifested as bile duct inflammation, fibrosis, and stenosis, which eventually progresses to liver cirrhosis and end-stage liver failure [54]. Its pathogenesis is not yet fully understood, and is believed to be driven by a combination of genetic susceptibility, environmental exposure, abnormal immune responses, bile acid homeostasis disturbance, and gut microbiota dysbiosis [55]. Unlike its protective role in MASLD, FGF1 plays a promotive role in PSC progression by regulating biliary response and liver fibrosis. Research has found that in a PSC mouse model, FGFR1-4 are upregulated in bile duct cells, which exhibit immunoreactivity to FGF1. Exogenous FGF1 exacerbates ductal response, liver inflammation, and fibrosis, while inhibition of the FGF1/FGFR signaling axis (via FGFR inhibitors or FGF1 monoclonal antibodies) effectively alleviates these pathological changes [56]. The underlying mechanism is that FGF1 upregulates the expression of Senescence-Associated Secretory Phenotype (SASP) and TGF-β1, which induces biliary proliferation/ductal response and biliary aging, and subsequently activates hematopoietic liver cells and liver fibrosis through paracrine mechanisms. Targeting the FGF1/FGFR signaling axis can reduce biliary aging, SASP release, and ductal response, thus mitigating PSC-related liver injury and fibrosis [56,57,58], indicating that this axis is a novel potential therapeutic target for PSC intervention.

Currently, PSC still lacks effective treatment methods, and combination drug therapy targeting multiple pathogenic aspects, including the FGF1/FGFR signaling axis, may provide new opportunities [59].

## 5. FGF1 and Alcohol-Related Liver Disease

Alcohol-related liver disease (ALD) is a common CLD caused by long-term heavy alcohol consumption, which progresses from alcoholic fatty liver and mild ALD to alcoholic hepatitis, liver fibrosis, and cirrhosis [60,61]. In 2021, the global number of ALD patients reached approximately 3.02 million (a 38.68% increase compared to 2000), the number of alcohol-related primary liver cancer patients was about 132,000 (a 94.12% increase compared to 2000), and the numbers of deaths from ALD and from alcohol-related primary liver cancer were 354,250 and 92,230, respectively (a 32.6% and a 76.75% increase compared to 2000) [62]. The complex pathogenesis of ALD involves multiple interrelated pathways including oxidative stress, abnormal lipid metabolism, endoplasmic reticulum stress, autophagy, multiple cell death modes, gut microbiota dysbiosis, and abnormal liver immune response [63]. At present, there are no direct experimental studies exploring the role of FGF1 in ALD. However, based on its proven ability to alleviate lipid accumulation in MASLD and DILI and its antioxidant biological function, we speculate that FGF1 may also play a role in ALD. However, the specific mechanism still requires further research in the future.

## 6. FGF1 and Liver Fibrosis

Liver fibrosis is a pathological repair process triggered by various chronic liver injuries, characterized by excessive accumulation of extracellular matrix (ECM) in liver tissue and progressive remodeling of hepatic architecture, ultimately leading to cirrhosis, liver failure, and even hepatocellular carcinoma [64]. The etiologies of liver fibrosis are diverse, including viral hepatitis, MASLD (particularly its progressive form MASH), ALD, cholestatic liver disease, and autoimmune liver disease. From an epidemiological perspective, CLD has become a global public health burden, with a nearly 13% increase in the number of affected individuals since 2000, and nearly all CLD progression is accompanied by liver fibrosis [64].

**Figure 2:**
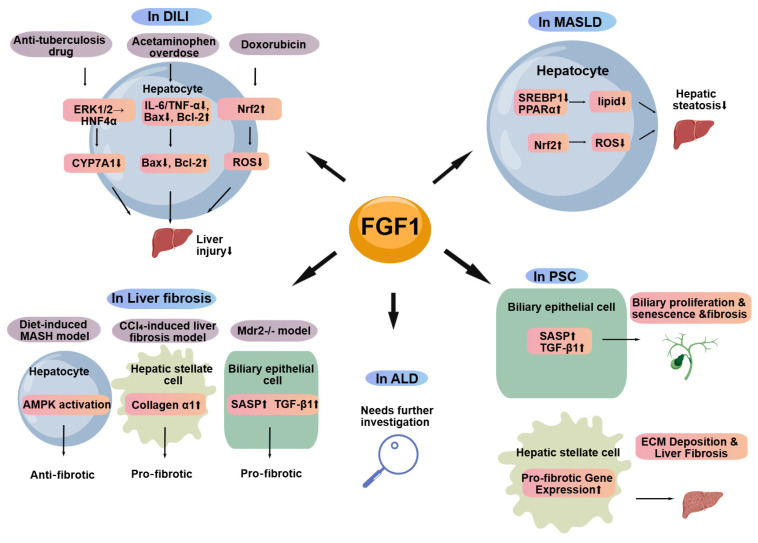
Schematic overview of FGF1-mediated regulatory networks in different chronic liver diseases.

In this context, members of the FGF family have garnered increasing attention for their roles in hepatic metabolism and fibrosis regulation. Classical metabolism-related FGFs, including FGF1, FGF19, and FGF21, have been shown to significantly ameliorate hepatic steatosis, inflammation, and fibrosis, positioning them as promising candidate molecules for the treatment of MASLD/MASH [65]. However, FGF1 exhibits highly specific and complex effects in liver fibrosis, displaying distinct actions depending on the disease etiology and, within the same model, on different hepatic cell types. In a mouse model of carbon tetrachloride (CCl_4_)-induced liver fibrosis, combined deletion of FGF1 and FGF2 significantly reduced collagen α1(I) synthesis and hepatic fibrotic deposition, suggesting that FGF1 promotes hepatic stellate cell (HSC)-driven matrix deposition in this model [66]. In primary sclerosing cholangitis (PSC), FGF1 acts as a pro-fibrotic factor in biliary epithelial cells [56]. In contrast, in a diet-induced MASH model, hepatocyte-derived FGF1 exerts anti-inflammatory and anti-fibrotic protective effects by activating the AMPK signaling pathway [67]. We propose that whether FGF1 exerts beneficial or detrimental effects depends on the cell type that dominates the pathological process, which may be attributed to the differential activation of downstream pathways upon FGFR stimulation in distinct cell types. This suggests that future research on FGF1-targeted therapies should fully consider the relevant cell types and tissue microenvironment and promote the development of tissue-specific targeted delivery strategies. To provide a concise overview, the key mechanisms of FGF1 in different types of CLD are summarized in Table 1 and Figure 2.

## 7. Discussion and Prospect

Some CLDs, such as ALD and anti-tuberculosis DILI, still lack FDA and EMA approved therapeutic drugs globally. The high prevalence of ALD and the clinical dilemma between liver injury treatment and anti-tuberculosis treatment make it crucial to search for new therapeutic targets and develop specific drugs. FGF1, as an important metabolic regulatory molecule, is expected to become a promising new target. Currently, FGF family members (FGF21, FGF19) have entered clinical trials, providing a valuable reference for the future development of FGF1-based CLD therapeutics. FGF1, FGF19, and FGF21 all belong to the same family, yet they differ fundamentally in receptor usage, co-factor dependence, metabolic function, and clinical translatability. FGF19 acts primarily as an endocrine factor via FGFR4 with β-Klotho as its obligatory co-receptor, regulating bile acid homeostasis, glycogen synthesis, and hepatocyte proliferation in an endocrine manner [68,69]. FGF21 is also β-Klotho-dependent, signaling through FGFR1/2/3 to modulate energy expenditure, insulin sensitivity, and hepatokine crosstalk [70,71]. In contrast, FGF1 is a classic paracrine factor that does not require β-Klotho and can activate all four FGFR subtypes [8]. This receptor pleiotropy enables FGF1 to regulate multiple signaling pathways, including PI3K/AKT, Ras/MAPK, and PLCγ, all of which are involved in metabolism, cell survival, proliferation, and tissue repair [72]. On the one hand, this broad activity endows FGF1 with pleiotropic effects extending beyond pure metabolic regulation—including anti-inflammatory activity, cytoprotection, and liver regeneration in acute injury models—but on the other hand, it raises mitogenic safety concerns that have hindered its clinical development compared with FGF19/21 [72]. By contrast, the β-Klotho-restricted signaling of FGF19 and FGF21 confers greater tissue specificity, leading to more predictable safety profiles in liver-focused applications. The unique value of FGF1 over FGF19/21 is its ability to activate all FGFR subtypes without β-Klotho dependence, thereby offering therapeutic potential for complex liver diseases that involve both metabolic injury and acute destruction.

At present, the potential pro-proliferative activity of FGF1 poses a carcinogenic risk, which represents a key challenge for its clinical translation. In one study, a recombinant FGF1 ligand lacking the first 24 N-terminal amino acid residues—designated FGF1Δ—was generated. This FGF1Δ exhibited significantly reduced binding activity to FGFRs compared with wild-type FGF1, retaining low affinity for FGFR1c and FGFR2c. The modified FGF1 showed reduced pro-proliferative activity in vitro, while its glucose-lowering effect was preserved. Notably, although FGF1 exerts its glucose-lowering effect through FGFR1, FGF1Δ retains a robust glucose-lowering function despite its low affinity for FGFR1c [51]. Formation of the FGF1–FGFR complex triggers multiple intracellular signaling pathways. Studies have identified that the glucose-lowering effect of FGF1 is primarily mediated through the PI3K/AKT pathway, whereas its pro-proliferative activity mainly relies on the Ras/MAPK pathway. Notably, the proliferative response requires strong and sustained FGFR signaling activation. This feature provides a viable strategy for the development of safer FGF1-based therapeutics: by introducing protein-level modifications that substantially reduce the binding activity of FGF1 to FGFRs, it is possible to attenuate its pro-proliferative activity while retaining weak affinity for a specific receptor subtype, thus preserving the activation of beneficial downstream signaling pathways associated with that receptor. A study on the development of FGF1 mutants further corroborated the validity of this strategy. By mutating the amino acid sequence, the researchers generated two FGF1 mutants—one with impaired FGFR binding and another with impaired heparin binding. Both mutants retained the glucose-lowering effect while exhibiting significantly reduced mitogenic activity [73]. Moreover, studies on the safety of FGF1 remain very limited to date. One cell-based study reported that the presence of FGF1 in vitro led to a marked increase in the proportion of human fibroblast-like cells exhibiting monosomy of chromosome 6, suggesting its potential carcinogenicity [74]. Furthermore, a clinical study involving plasmid-mediated FGF1 expression for the treatment of 93 patients with peripheral arterial ischemia reported that, over a three-year post-treatment follow-up, no statistically significant increase was observed in adverse events associated with angiogenic therapy—such as retinopathy, renal dysfunction, or cancer—following intramuscular injection of the FGF1-expressing plasmid. However, the study noted that the conclusions were limited by the small sample size. At the end of follow-up, two cases of cancer occurred in the plasmid treatment group, whereas no cancer cases were reported in the control group, which may serve as a warning signal. Thus, additional data are needed to draw reliable conclusions regarding safety [75]. FGF1 lacks safety studies involving long-term in vivo administration in animals; most articles have only discussed its risks at a theoretical level. Future research should focus on the long-term safety of FGF1 in animal models through experimental studies to determine whether the use of FGF1 significantly increases cancer risk and pay attention to the pathways through which FGF1 exerts its effects in different diseases, so that FGF1 can be rationally modified accordingly.

Aside from the potential carcinogenic risk associated with its pro-proliferative activity, the widespread distribution of FGFRs across many tissues and the short half-life of FGF1 in vivo present further challenges. Simple peripheral administration, on the one hand, struggles to achieve sufficient FGF1 accumulation at the target organ, and on the other, may induce adverse effects in certain tissues. Consequently, improving the tissue targeting of FGF1-based therapies is a critical issue that needs to be addressed. In a peripheral arterial ischemia trial, intramuscular injection of a plasmid expressing FGF1 was shown to confine FGF1 expression to the vicinity of the injection site while retaining its full biological activity [76]. In the liver, researchers employed an adeno-associated virus serotype 8 (AAV8) vector carrying the thyroxine-binding globulin promoter to achieve hepatocyte-specific expression of FGF1ΔHBS, which markedly alleviated hepatic steatosis, inflammation, and fibrosis without inducing hepatocyte hyperproliferation [77]. These studies suggest that for peripheral organ delivery of FGF1, engineering organ- or tissue-specific vectors to enable targeted and sustained self-expression of FGF1 in the target cells is a viable approach to enhance FGF1 targeting. In the liver, this strategy also effectively addresses the current challenge where FGF1 exerts protective effects on hepatocytes while promoting disease progression in hepatic stellate cells and cholangiocytes.

With the continuous deepening of research, FGF1 is expected to provide a new direction for the precise treatment of CLDs and bring new hope for patients with these currently difficult-to-treat liver diseases.

## Figures and Tables

**Table 1:** Mechanisms of FGF1 in different types of chronic liver disease.

Disease Category	Specific Cause/Model	Target Cell	Role of FGF1	Main Findings	Key Pathways/Targets	Potential Therapeutic Strategy
Drug-Induced Liver Injury	Anti-tuberculosis drugs (Isoniazid + Rifampicin)	Hepatocyte	Protective	Restores bile acid homeostasis; suppresses bile acid synthesis enzymes	FGFR4-ERK1/2-HNF4α → downregulates CYP7A1	FGF1 supplementation (protein or gene delivery); optimize dosing to avoid mitogenic risk
	Acetaminophen overdose	Hepatocyte	Protective	Anti-inflammatory, anti-apoptotic, alleviates oxidative and ER stress	Inhibits IL-6/TNF-α; downregulates Bax, upregulates Bcl-2	FGF1 as alternative or adjunct to NAC; requires half-life extension strategies
	Doxorubicin (DOX)	Hepatocyte	Protective	Activates antioxidant system Reduces oxidative stress and apoptosis	Activates Nrf2 → increases antioxidant enzymes	FGF1 combined with resveratrol; antioxidant synergy
Metabolic-Associated Fatty Liver Disease	High-fat diet/metabolic syndrome models	Hepatocyte	Protective	Suppresses lipogenesis, promotes fatty acid oxidation; Alleviates oxidative stress	Downregulates SREBP1, upregulates PPARα Promotes Nrf2 nuclear translocation.	FGF1ΔHBS with AAV8-mediated hepatocyte-specific delivery; combination with metabolic drugs
Primary Sclerosing Cholangitis	Mdr2−/− model	Biliary epithelial cell	Disease-promoting	Induces cholangiocyte senescence, ductular reaction, and activates hepatic stellate cells	FGF1/FGFR → upregulates SASP and TGF-β1 →Biliary proliferation & senescence & fibrosis	FGFR inhibitors or FGF1-neutralizing antibodies; anti-senescence strategies
		Hepatic stellate cell	Disease-promoting	Pro-fibrotic Gene Expression	ECM Deposition & Liver Fibrosis	
Alcohol-related liver disease (need future research)	-	Maybe hepatocyte	Maybe protective	-	-	
Liver fibrosis	Mdr2−/− model	Biliary epithelial cell	Disease-promoting	Induces cholangiocyte senescence, ductular reaction, and activates hepatic stellate cells	FGF1/FGFR → upregulates SASP and TGF-β1 →Biliary proliferation & senescence & fibrosis	FGFR inhibitors or FGF1-neutralizing antibodies; anti-senescence strategies
	CCl_4_-induced liver fibrosis model	Hepatic stellate cell	Disease-promoting	FGF1-induced upregulation of collagen α1 expression in HSCs	Promote liver fibrosis	HSC-specific FGFR inhibition; avoid global FGFR blockade
	Diet-induced MASH model	Hepatocyte	Protective	AMPK activation	Anti-inflammatory and fibrotic	Hepatocyte-specific FGF1 delivery (e.g., AAV8-FGF1ΔHBS)

## Data Availability

No new data were created or analyzed in this study.
